# Yunnan Baiyao Conditioned Medium Promotes the Odonto/Osteogenic Capacity of Stem Cells from Apical Papilla via Nuclear Factor Kappa B Signaling Pathway

**DOI:** 10.1155/2019/9327386

**Published:** 2019-04-23

**Authors:** Xiyao Pang, Yanqiu Wang, Jintao Wu, Zhou Zhou, Tao Xu, Lin Jin, Yan Yu, Zehan Li, Romila Gobin, Changao Xue, Jinhua Yu

**Affiliations:** ^1^Department of Stomatology, Nanjing First Hospital, Nanjing Medical University, Nanjing, Jiangsu, China; ^2^Key Laboratory of Oral Diseases of Jiangsu Province and Stomatological Institute of Nanjing Medical University, Nanjing, Jiangsu, China; ^3^Endodontic Department, School of Stomatology, Nanjing Medical University, Nanjing, Jiangsu, China; ^4^Department of Stomatology, Nanjing Central Hospital, Nanjing, Jiangsu, China; ^5^Endodontic Department, Nantong Stomatological Hospital, Nantong, Jiangsu, China

## Abstract

Yunnan Baiyao is a traditional Chinese herbal remedy that has long been used for its characteristics of wound healing, bone regeneration, and anti-inflammation. However, the effects of Yunnan Baiyao on the odonto/osteogenic differentiation of stem cells from apical papilla (SCAPs) and the potential mechanisms remain unclear. The aim of this study was to investigate the odonto/osteogenic differentiation effects of Yunnan Baiyao on SCAPs and the underlying mechanisms involved. SCAPs were isolated and cocultured with Yunnan Baiyao conditioned media. The proliferation ability was determined by cell counting kit 8 and flow cytometry. The differentiation capacity and the involvement of NF-*κ*B pathway were investigated by alkaline phosphatase assay, alizarin red staining, immunofluorescence assay, real-time RT-PCR, and western blot analyses. Yunnan Baiyao conditioned medium at the concentration of 50 *μ*g/mL upregulated alkaline phosphatase activity, induced more mineralized nodules, and increased the expression of odonto/osteogenic genes/proteins (e.g.,* OCN*/OCN,* OPN*/OPN,* OSX*/OSX,* RUNX2*/RUNX2,* ALP*/ALP,* COL-I*/COL-I, DMP1,* DSP*/DSPP) of SCAPs. In addition, the expression of cytoplasmic phos-I*κ*B*α*, phos-P65, and nuclear P65 was significantly increased in Yunnan Baiyao conditioned medium treated SCAPs in a time-dependent manner. Conversely, the differentiation of Yunnan Baiyao conditioned medium treated SCAPs was obviously inhibited when these stem cells were cocultured with the specific NF-*κ*B inhibitor BMS345541. Yunnan Baiyao can promote the odonto/osteogenic differentiation of SCAPs via the NF-*κ*B signaling pathway.

## 1. Introduction

Immature permanent teeth with necrotic pulps have always been a challenge to many endodontists [1]. Dental caries, trauma, or abnormal central cusp can lead to the bacterial invasion, resulting in irreversible pulpitis, necrosis, and the cessation of root development [2]. Traditionally, calcium hydroxide and mineral trioxide aggregate (MTA) are being used to induce the pulp revascularization or apexification at the root apex [3, 4]. Previous histological studies have indicated that MTA stimulates tissue regeneration and bone repair. Dentinogenesis is induced more effectively in MTA group than calcium hydroxide group [5]. However, there is still the risk of high cytotoxicity, weakened radicular dentin, horizontal root fracture, especially at the cervical area, and crown discoloration [6]. Some categories including MTA and ZnOE are capable of inducing intrinsic staining after interacting with dentin, causing an alteration of the outward appearance of the tooth [7, 8]. Therefore, exploring new ways to trigger pulp regeneration and preserve pulp vitality is of great importance.

Traditional Chinese medicine (TCM) plays a significant role in the treatment of tooth ache, bone fracture, and osteoarthritis in many oriental countries for a very long lapse of time [9, 10]. Yunnan Baiyao (YNB), which was formulated in 1902, has complex components including radix notoginseng, forest musk, radix aconiti kusnezoffii, and rhizoma paridis chonglou [11, 12]. It is well-known in China for its numerous clinical applications such as vasodilation, anti-inflammation, wound healing, and regeneration of damaged bones [13]. Modern pharmacological studies have revealed that YNB contains multiple active components including the calcium ion which is important for cell proliferation and differentiation [14].

Stem cells from apical papilla (SCAPs) are a kind of ectomesenchyme-derived cells encased by the dentin tissue during root formation [15, 16]. Because of the collateral circulation at the apical location, SCAPs are able to survive when immature permanent teeth undergo pulp necrosis or periapical inflammation [17]. As compared to other mesenchymal cells which include periodontal ligament stem cells (PDLSCs), dental pulp stem cells (DPSCs), and bone marrow stem cells (BMSCs), the postnatal stem cells have higher proliferation rates and odontogenic differentiation capacity [18, 19]. There is also evidence of their ability to form tooth root-like structures in animal models [20]. In minipig models, the utilization of SCAPs and biological scaffolds like hydroxyapatite/tricalcium phosphate (HA/TCP) in sockets of the lower jaw can generate dentin-like tissues [21]. Therefore, SCAPs are considered to be an attractive candidate for pulp regeneration and bioroot engineering.

The fact that YNB shows ideal effects on the repair of broken bones prompts us to consider whether it might have an effect on dental pulp tissues. This study aims to take the first step in evaluating the differentiation ability of YNB against SCAPs* in vitro*. For this reason, SCAPs were cultured in YNB conditioned media (YNB-CM). The proliferation and odonto/osteogenic differentiation of SCAPs were subsequently evaluated* in vitro*. Our findings indicated that YNB can be used as a potential drug to regenerate the dentin-pulp complex and induce continued root development.

## 2. Materials and Methods

### 2.1. X-Ray Powder Diffraction (XRD)

The YNB powders were obtained by grinding with the aid of an agate mortar and pestle for 15 minutes. The resultant powders were then dusted through a sieve of 360 mesh directly on a holder and illuminated with x-rays by energy dispersive spectrometer (EDS) (Noran, USA). X-ray was used to probe the structures of YNB powders. The peaks of solid solution were measured with a peak search algorithm [22, 23]. Each peak in the diffraction pattern corresponds to a set of unique crystallographic planes.

### 2.2. Cell Isolation and Culture

Human impacted noncarious third molars (n=21) were harvested after obtaining informed consent from young patients (15-20 years old) in Oral Surgery Department of Jiangsu Provincial Stomatological Hospital. The study was approved by Ethical Committee of the Stomatological School of Nanjing Medical University (reference no. IACUC1601026). The apical papillae were gently separated with a pair of tweezers, manually scraped and enzymatically digested for 30 minutes at 37°C. Then, cells were purified using rabbit anti-STRO-1 antibody (Santa Cruz, Delaware, CA) and sheep anti-rabbit IgG Dynabeads (Dynal Biotech, Oslo, Norway) followed by magnetic activated cell sorting (MACS) instructions. Isolated cells were then cultured in alpha minimum essential medium (*α*-MEM, Gibco, Life Technologies, Grand Island, NY, USA) supplemented with 10% fetal bovine serum (FBS, Gibco, USA), 100 U/ML penicillin-streptomycin (Sigma-Aldrich, St. Louis, MO, USA) at 37°C in 5% CO2. Based on cell surface markers, SCAPs were identified by flow cytometry as previously described [24, 25]. Once reached 80-90% confluence, cells were passaged after trypsinization. Subsequent experiments were performed using SCAPs at 3-5 passages.

### 2.3. Preparation of Yunnan Baiyao Conditioned Medium

Yunnan Baiyao (Yunnan Baiyao Group Co., Ltd., Kunming, China) was ground into fine powders. The powders were filtered through a 45 *μ*m strainer and condensed to 2 mg/mL using *α*-MEM. The solution was vortexed until completely suspended and incubated for 1 week at 37°C to obtain the bioactive contents of YNB [26, 27]. The supernatant was filtered through a 0.22 *μ*m strainer (Corning, NY, USA) before use. The solution was stored at −20°C in dark. Cells were refreshed every 48 hours with prepared YNB conditioned media.

### 2.4. Cell Counting Kit 8 (CCK8) Assay

A density of 2 × 10^3^ cells per well was seeded into 96-well plates (Corning, NY, USA). After 24 hours of culture, the media were replaced with YNB conditioned media at different concentrations. After 1, 3, 5, 7, and 9 days of culture, CCK8 reagents (CCK8, Dojindo, Japan) were added to each well and incubated for 2 hours at 37°C. The absorbance was measured at the wave length of 490 nm.

### 2.5. Flow Cytometry for Cell Cycle and Cell Apoptosis

For cell cycle analysis, cells were trypsinized, harvested, and then fixed with 75% ice-cold ethanol at 4°C for 30 min in the dark. DNA content was determined by FAC-Scan flow cytometer (BD Biosciences, San Jose, CA). For the detection of cell apoptosis, cells were collected by trypsin and rinsed with 0.01 mol/L phosphate buffer saline (PBS) twice. The cells were then detected by flow cytometry with an Annexin V-FITC/PI apoptosis detection kit (Dojindo, Kumamoto, Japan) according to the manufacturer's instructions.

### 2.6. Alkaline Phosphatase (ALP) Activity and Staining

SCAPs were plated into 6-well plates (Nunc, USA) at a concentration of 1 × 10^4^ cells/well and cultured in YNB-CM at the concentrations of 0, 20, 50, 100, and 200 *μ*g/mL, respectively. ALP activities and protein concentrations of each group were, respectively, measured at day 3 and day 5 with a bicinchoninic acid (BCA) protein assay kit (Beyotime, China) and an ALP activity kit (Jiancheng, China) according to the manufacturer's manual. ALP activity was calculated after normalization to the total protein content. Subsequently, YNB-treated SCAPs at different concentrations were cultured in 96-well pates at 3000 cells/wells. Cells were fixed at day 3 and ALP staining was assessed following the manufacturer's protocol (Beyotime, China). According to ALP levels, 50 *μ*g/mL was selected as the optimal concentration for the subsequent experiments.

### 2.7. Alizarin Red Staining

For alizarin red staining (ARS), SCAPs were seeded into 24-well plates at a density of 2 × 10^4^ cells/well and grown to subconfluence. After incubation for 24 hours, the medium was replaced with four different media (complete medium, 50 *μ*g/mL YNB conditioned medium, MM, MM+50 *μ*g/mL YNB conditioned medium) for 2 weeks. MM, also called mineralization-inducing media, is supplemented with 10 mmol/L *β*-glycerophosphate (Sigma-Aldrich), 50 mg/L ascorbic acid (Sigma-Aldrich), and 10 nmol/L dexamethasone (Sigma-Aldrich). Alizarin red staining and the quantification of mineralization were carried out at day 14 as described before and a scanner was used to take images [28]. To quantify the degree of mineralization, the alizarin red was eluted by 10% cetylpyridinium chloride (CPC, Sigma-Aldrich) in 10 mmol/L sodium phosphate for 30 min at room temperature. The absorbance of the calcium contents was measured at 562 nm with a universal microplate reader (BioTek Instruments, USA). Alizarin red staining intensity was calculated after normalization to the total protein content.

### 2.8. Immunofluorescence Assay

Cells treated by YNB conditioned media were cultured on sterile glass coverslips. After 3 days of culture, cells were fixed with 4% paraformaldehyde for 30 minutes, permeabilized with 0.5% Triton X-100 for 10 minutes and then blocked with goat serum for 30 minutes at room temperature. After that, an incubation with primary antibodies including runt-related transcription factor 2 (RUNX2, 1:100, Abcam), type I collagen (COL-I, 1:100, Abcam), P65 (1:100, Cell Signaling Technology), I*κ*B*α* (1:100, Cell Signaling Technology) was performed overnight at 4°C. The cells were subsequently washed with PBS for three times and incubated with a secondary antibody in the dark for 1 hour. Nuclei were then counterstained with 4.6-diamidino-2-phenylindole (DAPI, 1:1,000, Invitrogen) for 2 minutes. Images were captured with the inverted fluorescence microscopy (Olympus, Japan).

### 2.9. Real-Time Reverse Transcription Polymerase Chain Reaction (Real-Time RT-PCR)

Total RNA was extracted from cells with TRIzol reagent (Invitrogen, Carlsbad, USA). Reverse transcription into complementary DNA was carried out using a PrimeScript RT Master Mix kit (TaKaRa Biotechnology, Dalian, China). Real-time RT-PCR was performed using SYBR Green Master (Roche, Indianapolis, IN, USA) and ABI 7300 real-time PCR system. Primer sequences were listed in Table 1. Glyceraldehyde-3-phosphate dehydrogenase (*GAPDH)* was served as the reference gene for normalization and the expression of osteo/odontoblastic genes including osteocalcin (*OCN*), osteopontin (*OPN*), osterix (*OSX*),* RUNX2*,* ALP*,* COL-I*, and dentin sialoprotein (*DSP*) was calculated by comparison Ct (2^−ΔΔCt^) method as previously reported [29]. All PCR assays were performed in triplicate to minimize the errors.

### 2.10. Western Blot Analysis

Cells pretreated with or without YNB conditioned media were respectively cultured for 3 and 7 days and then collected. Cells were lysed in radioimmunoprecipitation assay (RIPA) lysis buffer (Beyotime, China) supplemented with proteinase inhibitors. The cytoplasmic and nuclear proteins were respectively extracted after cells were exposed to YNB for 0, 15, 30, 60, and 120 minutes with a Keygen Kit. The kit enables the stepwise lysis of cells and the separation of the cytoplasm from the intact nuclease as well as the extraction of nuclear proteins away from genomic DNA and mRNA. 20 *μ*g proteins per well were loaded onto a 5% stacking and 12% resolving SDS-PAGE gel and electrotransferred onto polyvinylidene fluoride (PVDF) microporous membranes (Millipore, Bedford, MA, USA). After blocking with 5% Bovine Serum Albumin (BSA), membranes were subsequently incubated with primary antibodies to detect OCN (1: 1000, Abcam), OPN (1:1000, Abcam), OSX (1:1000, Abcam), RUNX2 (1:1000, Abcam), ALP (1:1000, Abcam), COL-I (1:1000, Abcam), dentin matrix protein 1 (DMP1,1:1000, Abcam), dentin sialophosphoprotein (DSPP,1:1000, Bioworld), phosphorylated I*κ*B*α* (1:1000, Cell Signaling Technology), I*κ*B*α* (1:1000, Cell Signaling Technology), phosphorylated p65 (1:1000, Cell Signaling Technology), P65 (1:1000, Cell Signaling Technology), Histone 3 (H3, 1:1000, Cell Signaling Technology), and GAPDH (1:1000, Bioworld) overnight. Finally, after washing with TBST, the membranes were reacted with a secondary antibody for 1 hour at room temperature, visualized and scanned by ImageQuant LAS 4000 system (GE Healthcare, USA).

### 2.11. Statistical Analysis

The quantitative results were graphed and analyzed as the means ± standard deviation (SD). One way analysis of variance (ANOVA) and Student's* t*-test was performed to determine statistical differences between groups. All experiments were replicated at least three times. All statistical calculations were performed with SPSS 22.0 software (SPSS Inc., Chicago, IL, USA).* P*<0.05 or <0.01 was considered as statistically significant.

## 3. Results

### 3.1. Screening of YNB Conditioned Media

**Figure 1:**
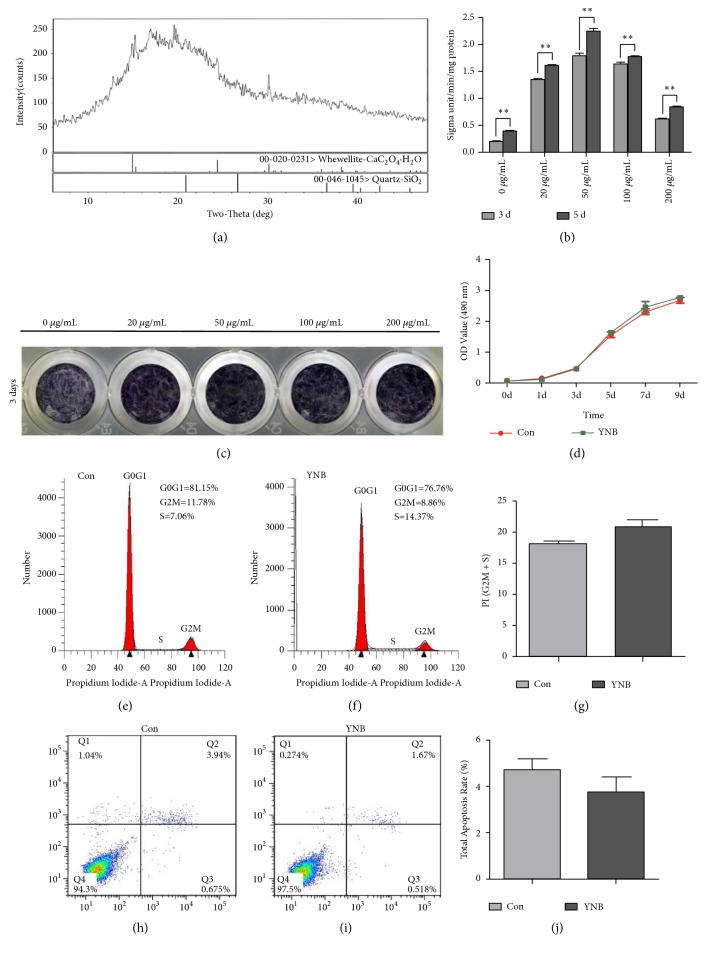
*XRD diffractogram of YNB and its effects on ALP activity and proliferation of SCAPs*. (a) XRD diffractogram for YNB powders. (b) ALP activity of SCAPs at day 3 and day 5 respectively, *∗∗P*<0.01. (c) Representative images of ALP staining at day 3. (d) CCK8 assay for 9 consecutive days, *∗∗P*<0.01. (e) Representative flow cytometry analysis of cell cycle in the control group. (f) Representative flow cytometry analysis of cell cycle in the YNB group. (g) Average proliferation index (PI) in the control group and YNB group. Values are means ± SD, n=3. (h) Representative flow cytometry analysis of cell apoptosis in the control group. (i) Representative flow cytometry analysis of cell apoptosis in the YNB group. (j) Total apoptosis rate in the control group and YNB group. Values are means ± SD, n=3.

The X-ray diffractogram demonstrated that the major crystal structure of YNB was CaC_2_O_4_·H2O (Figure 1(a)). To investigate the effects of YNB on the ALP activity, SCAPs were treated with different concentrations of YNB-CM for 3 days and 5 days, respectively, 20-200 *μ*g/mL YNB-CM elevated the ALP activity of SCAPs as compared with the untreated group, in which 50 *μ*g/mL YNB-CM treated cells showed the highest ALP activity among all groups (Figure 1(b)). Furthermore, ALP positive staining was also significantly upregulated in 50 *μ*g/mL group (Figure 1(c)). Therefore, 50 *μ*g/mL was selected as the optimal concentration in this study and used for the subsequent experiments. The CCK8 assay was performed for 9 consecutive days. The results demonstrated no significant difference between 50 *μ*g/mL group and the control group (Figure 1(d)). Flow cytometry analysis revealed that 50 *μ*g/mL YNB exerted almost no effect on the proliferation and apoptosis of SCAPs (Figures 1(e), 1(f), 1(h), and 1(i)). There were no statistically significant difference (*P*>0.05) in the proliferation index and apoptosis index between the control group and YNB group (Figures 1(g) and 1(j)).

### 3.2. YNB Induced the Odonto/Osteogenic Differentiation of SCAPs

**Figure 2:**
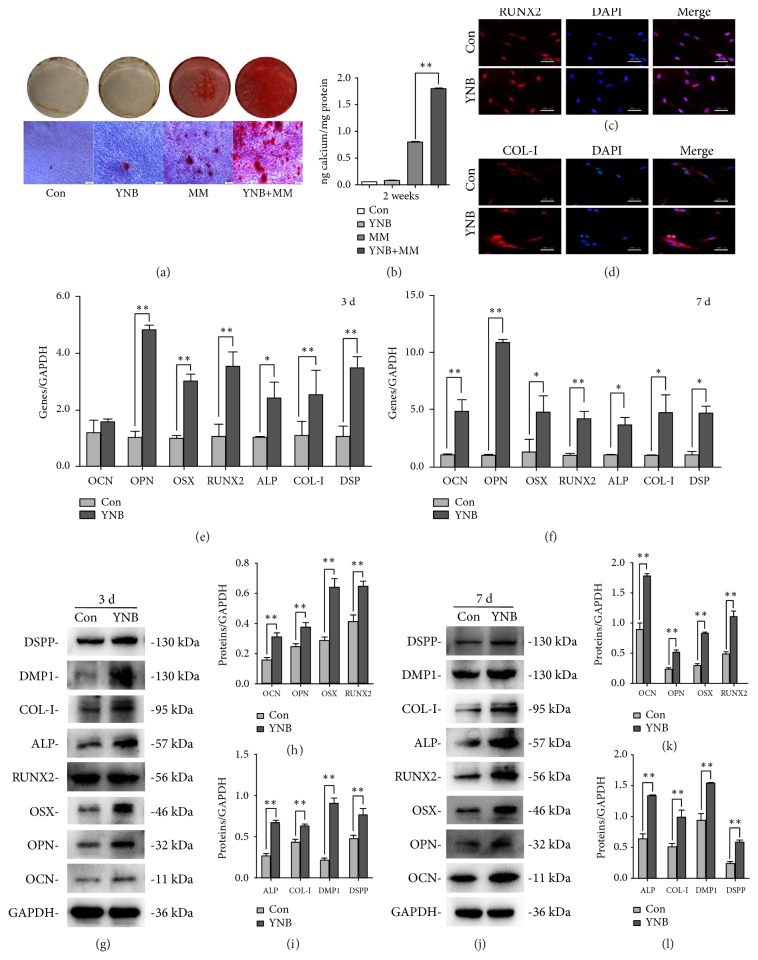
*Effects of YNB on odonto/osteogenic differentiation of SCAPs*. (a) Representative images of ARS and calcium nodules under the inverted microscope (scale bars = 200 *μ*m). (b) Quantitative analyses of calcium nodules. Values are means ± SD, n=3, *∗∗P*<0.01. (c) Immunofluorescent staining of RUNX2 in the control group and YNB group. Scale bar = 200 *μ*m. (d) Immunofluorescent staining of COL-I in the control group and YNB group. Scale bar = 200 *μ*m. (e, f) mRNA expressions of* OCN*,* OPN*,* OSX*,* RUNX2*,* ALP*,* COL-I,* and* DSP* in different groups by real-time RT-PCR at day 3 and day 7. Values are described as means ±SD, n=3. *∗∗*2^−ΔΔCt^ > 2,* P*<0.01; *∗*1 < 2^−ΔΔCt^ < 2,* P*<0.05. (g, j) The expression levels of the odonto/osteogenic proteins in the control group and YNB group at day 3 and day 7, respectively. (h, i) Grayscale analysis of the odonto/osteogenic markers at day 3. n=3. *∗P*<0.05, *∗∗P*<0.01. (k, l) Grayscale analysis of the odonto/osteogenic markers at day 7. n=3. *∗P*<0.05, *∗∗P*<0.01.

To elucidate the effects of YNB on the odonto/osteogenic differentiation of SCAPs, SCAPs were cultured in routine media or osteogenic differentiation medium containing YNB. Enhanced mineralization in YNB-treated SCAPs was shown by alizarin red staining as compared with control group (*P*<0.05) after 2 weeks of induction (Figures 2(a) and 2(b)). The immunofluorescent results demonstrated that the expression of COL-I and RUNX2 in YNB-treated SCAPs were upregulated in both nuclei and cytoplasm as compared with the control group (Figures 2(c) and 2(d)). Moreover, the odonto/osteogenic differentiation markers (e.g., OCN, OPN, OSX, RUNX2, ALP, COL-I, DMP1, DSPP) were significantly upregulated in YNB-treated SCAPs both at mRNA and protein levels after 3 or 7 days coculture (*P*<0.05, Figures 2(e)–2(l)).

### 3.3. YNB Caused the Activation of NF-*κ*B Pathway in SCAPs

**Figure 3:**
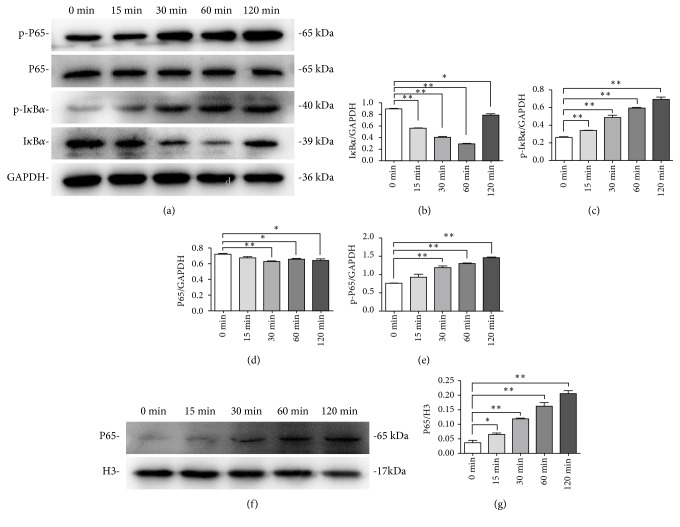
*Effects of YNB on NF-κB signaling pathway of SCAPs*. (a) The expression levels of I*κ*B*α*, p-I*κ*B*α*, P65, and p-P65 in the cytoplasm of YNB-treated SCAPs at the indicated times. GAPDH served as a loading control. (b-e) Grayscale analyses of I*κ*B*α*, p-I*κ*B*α*, P65, and p-P65. n=3. *∗P*<0.05, *∗∗P*<0.01. (f) The expression levels of nuclear P65 in YNB-treated SCAPs at the indicated times. Histone 3 served as a loading control. (g) Grayscale analysis of P65 in nuclei. n=3. *∗P*<0.05, *∗∗P*<0.01.

To evaluate the potential involvement of NF-*κ*B signaling pathway in YNB-treated SCAPs, the cytoplasmic and nuclear proteins at 0, 15, 30, 60, 120 min were, respectively, collected and the indicated protein levels were determined by western blot (Figures 3(a)–3(e)). Phosphorylated I*κ*B*α* was obviously elevated in YNB-treated SCAPs in a time-dependent manner while the expression of cytoplasmic I*κ*B*α* degraded rapidly during the first 60 minutes. Furthermore, the phosphorylated level of P65 was gradually elevated from 0 min to 120 min. In addition, western blot analysis showed a rapid and sustained increase of nuclear P65 expression in YNB-treated SCAPs (Figures 3(f) and 3(g)).

### 3.4. Inhibition of NF-*κ*B Pathway Suppresses the Odonto/Osteogenic Differentiation of YNB-Treated SCAPs

**Figure 4:**
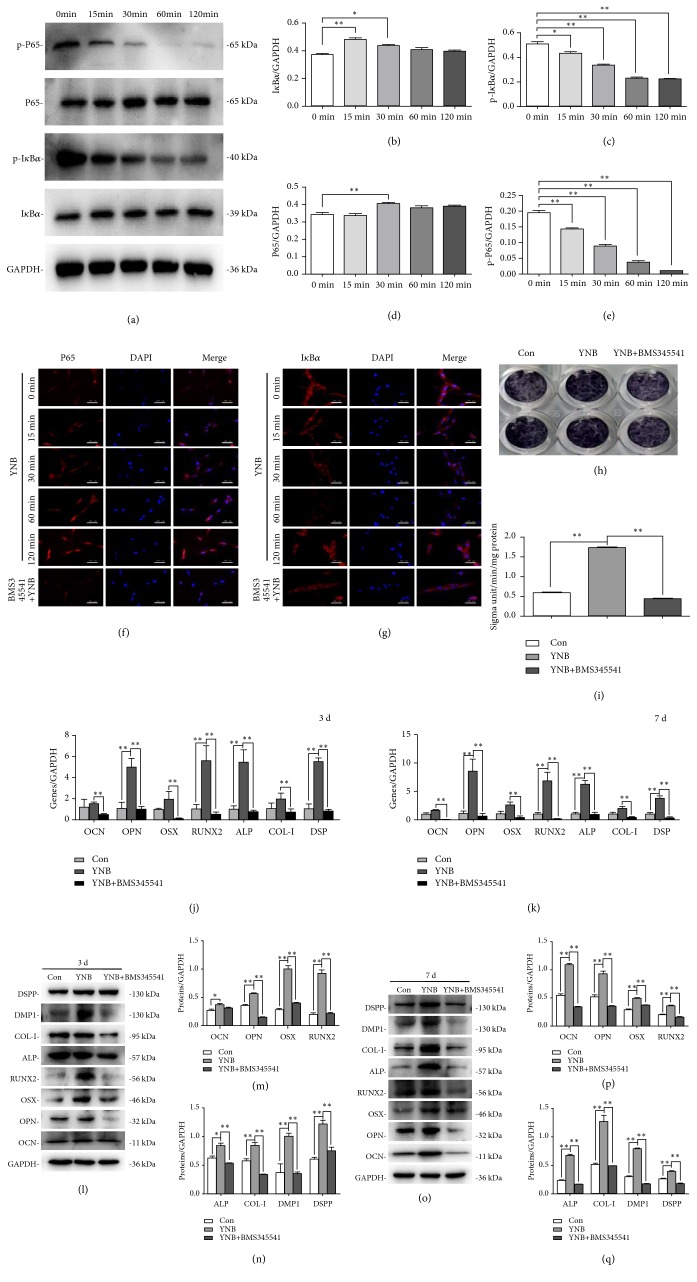
*Effects of cotreatment by YNB and NF-κB inhibitor BMS345541 on the odonto/osteogenic differentiation of SCAPs*. (a) The protein levels of cytoplasm I*κ*B*α*, p-I*κ*B*α*, P65, and p-P65 in YNB-treated SCAPs at different time points. GAPDH served as a loading control. (b-e) Semiquantitative analyses of cytoplasmic I*κ*B*α*, p-I*κ*B*α*, P65, and p-P65. n=3. *∗P*<0.05, *∗∗P*<0.01. (f, g) Immunofluorescence staining of P65 and I*κ*B*α* in YNB-CM treated SCAPs at 0, 15, 30, 60, 120 min and with NF-*κ*B inhibitor BMS345541, respectively. Scale bar = 200 *μ*m. (h, i) ALP activity and ALP staining in BMS345541 + YNB group and YNB group after 3 days of culture. Values are means ± SD, n=3. *∗P*<0.05, *∗∗P*<0.01. (j, k) mRNA expression of OCN, OPN, OSX, RUNX2, ALP, COL-I, and DSP in different groups by real-time RT-PCR after cotreatment with YNB and BMS345541 at day 3 and day 7, respectively. *∗*2^−ΔΔCt^≧2, n=3. (l, o) The expression levels of the odonto/osteogenic proteins in different groups after cotreatment with BMS345541 and YNB at day 3 and day 7, respectively. Values are means ± SD, n=3. *∗P*<0.05, *∗∗P*<0.01. (m, n) Grayscale analyses of the odonto/osteogenic proteins at day 3. *∗P*<0.05, *∗∗P*<0.01. (p, q) Grayscale analyses of the odonto/osteogenic proteins at day 7. *∗P*<0.05, *∗∗P*<0.01.

To further confirm the role of individual NF-*κ*B in the odonto/osteogenic differentiation of YNB-treated SCAPs, BMS345541 (an IKK inhibitor) was used to block the NF-*κ*B signaling pathway. BMS345541 remarkably reduced the phosphorylation of I*κ*B*α* and P65, inhibiting YNB-induced degradation of I*κ*B*α* and nuclear translocation of P65 (Figures 4(a)–4(e)). Immunofluorescence staining revealed that a rapid degradation of cytoplasmic I*κ*B*α* was synchronized with the translocation of P65 to the nuclei in a time-dependent manner (Figures 4(f) and 4(g)). In addition, when cotreated with BMS345541, the odonto/osteogenic genes (*OCN*,* OPN, OSX, RUNX2, ALP, COL-I,* and* DSP*) were not increased at different time points (Figures 4(j) and 4(k)). Western blot results and ALP activity assay further verified the above findings (Figures 4(h)–4(i) and 4(l)–4(q)).

## 4. Discussion

YNB, a mixture of various herbs and plants, has been long renowned for its clinical functions in bone regeneration and hemostasis [30]. Some studies have suggested that YNB can effectively promote the cell spreading of Caco-2/BBE cells and the committed differentiation of periodontal ligament stem cells (PDLSCs) [31]. In the current study, YNB conditioned medium had no effect on cell apoptosis and proliferation of SCAPs, as indicated by CCK8 and FCM results. However, YNB conditioned media can upregulate the expression of several osteo/odonto markers and increase the ratio of ARS/ALP positive cells, suggesting that YNB can promote the odonto/osteoblastic differentiation of SCAPs.

In detail, DSP, DSPP, and DMP1 are responsible for dentin formation and the precisely oriented hydroxyapatite crystals initiation in calcifying matrices [32]. DSPP may act as a downstream effector molecule of DMP1 during dentinogenesis. Long bone defects in DMP1 knockout mice can be partially rescued by exogenous DSPP [33]. Su-Jin Park et al. have reported that the expression of DSPP in early amelogenesis is linked to CPNE7, a preameloblast-derived factor correlated with odontoblast differentiation [34]. RUNX2 serves as an essential transcription factor for dental mineralization, skeletal generation, and bone matrix production [35]. OSX, a downstream factor of RUNX2, also plays a paramount role in the functional odonto/osteoblasts [36]. The impaired bone formation owing to inactivation of OSX results in perinatal lethality in mice [37]. ALP measurement is considered as an early marker of osteogenic differentiation whereas OPN and OCN usually reflect the endpoint of osteogenesis* in vitro* [38]. COL-I is broadly distributed in bone and dentin, which acts as a structural support and biological signal to surrounding cells [39]. In the present study, upregulated odonto/osteogenic markers of both early-stage and late-stage indicated the long term effects of YNB on the committed differentiation of SCAPs.

Nuclear factor kappa B pathway plays a crucial and evolutionary conserved role in skeletal development, tooth organogenesis, the modifications of mesenchymal stem cells, and eruption process. In many cell types, NF-*κ*B activation is essential for the expression of a wide variety of genes. Some studies have shown that interferon-*γ* (IFN-*γ*) can regulate human dental pulp stem cells behavior via NF-*κ*B and MAPK signaling [40]. Tumor necrosis factor *α* (TNF*α*) can promote the proliferation and osteogenesis of adipose-derived stem cells (ADSCs) through NF-*κ*B pathway [41]. Apart from cytokines, MTA, a specialized dental cement, can also trigger the odonto/osteogenic differentiation of inflammatory DPSCs via NF-*κ*B signaling pathway [42]. Our findings further proved that YNB can increase the phosphorylation of I*κ*B*ɑ* and P65, thus enabling the translocation of NF-*κ*B dimmers into the nucleus, which then binds with DNA to activate the subsequent transcription. Conversely, YNB-mediated differentiation of SCAPs was dramatically suppressed and NF-*κ*B signaling pathway was negatively regulated by BMS345541 (an NF-*κ*B IKK inhibitor), as indicated by the downregulated expression of odonto/osteogenic genes/proteins and decreased ALP activity. The above findings revealed that YNB can promote the odonto/osteogenic differentiation of SCAPs by activating the NF-*κ*B pathway.

The XRD results demonstrated the presence of rich calcium ions in YNB powders. Calcium ions allow the secretion of hemostatic agent in contact with interstitial fluids and therefore activates thrombocytes [43]. Besides, when calcium ion is released into the microenvironment, it interacts with various cytokines, growth factors, neuropeptides, and so on, stimulating the formation of bones and hard tissues [44]. It is believed that the potential pharmaceutical mechanism of YNB on the odonto/osteogenic differentiation is at least partially due to the excretion of calcium ions, which subsequently results in the accumulation of IKKs and ultimately leads to gene expression and protein synthesis that is related to the committed differentiation of SCAPs [45, 46]. Additionally, inflammatory cytokines including TNF*α* and interleukin-1 (IL-1) triggered by carious lesions or dental injuries in the dental pulp can be interrupted by YNB via NF-*κ*B signaling pathway, which in turn promote the odonto/osteogenic differentiation capacity of SCAPs [47].

However, these results are mainly based on intracellular molecular alterations and do not take into account the* in vivo* changes. The upregulation of several osteo/odontogenic markers does not necessarily mean that the YNB can actually induce* in vivo* pulp regeneration. The* in vivo* test and clinical application is also necessary for strengthening the current data to reach a better endodontic practice.

## 5. Conclusion

In summary, YNB conditioned medium can induce the odonto/osteogenic differentiation of SCAPs via NF-*κ*B signaling pathway from the molecular level, which may create a potent inductive microenvironment necessary for the upcoming pulp regeneration and apexogenesis. Definitely, further studies are required to explore the potential* in vivo* effects of YNB as well as clinical applications in endodontic practice, which may help us better understand the network controlling these processes.

## Figures and Tables

**Table 1:** Sense and antisense primers for real-time reverse transcription polymerase chain reaction.

Genes	Primers	Sequences (5′-3′)
*DSP*	Forward	ATATTGAGGGCTGGAATGGGGA
	Reverse	TTTGTGGCTCCAGCATTGTCA
*COL-I*	Forward	CCCTTTCTGCTCCTTTCT
	Reverse	TGTTTCCTGTGTCTTCTGG
*ALP*	Forward	GACCTCCTCGGAAGACACTC
	Reverse	TGAAGGGCTTCTTGTCTGTG
*RUNX2*	Forward	TCTTAGAACAAATTCTGCCCTTT
	Reverse	TGCTTTGGTCTTGAAATCACA
*OSX*	Forward	CCTCCTCAGCTCACCTTCTC
	Reverse	GTTGGGAGCCCAAATAGAAA
*OPN*	Forward	CCTGACTATCAATCACATCGGAAT
	Reverse	TGACCAGAGTGCTGAAACCCA
*OCN*	Forward	AGCAAAGGTGCAGCCTTTGT
	Reverse	GCGCCTGGGTCTCTTCACT
*GAPDH*	Forward	GAAGGTGAAGGTCGGAGTC
	Reverse	GAGATGGTGATGGGATTTC

## Data Availability

The data used to support the findings of this study are available from the corresponding author upon request. The readers can contact Professor Yu via email (email address: yujinhua@njmu.edu.cn) to obtain data.

## References

[1] Bose R., Nummikoski P., Hargreaves K. (2009). A retrospective evaluation of radiographic outcomes in immature teeth with necrotic root canal systems treated with regenerative endodontic procedures. *Journal of Endodontics*.

[2] Sheiham A., James W. P. T. (2015). Diet and dental caries: the pivotal role of free sugars reemphasized. *Journal of Dental Research*.

[3] Aksel H., Askerbeyli-örs S., Deniz-Sungur D. (2017). Vertical root fracture resistance of simulated immature permanent teeth filled with MTA using different vehicles. *Journal of Clinical and Experimental Dentistry*.

[4] Gokturk H., Bayram E., Bayram H. M., Aslan T., Ustun Y. (2017). Effect of double antibiotic and calcium hydroxide pastes on dislodgement resistance of an epoxy resin-based and two calcium silicate-based root canal sealers. *Clinical Oral Investigations*.

[5] Gandolfi M. G., Ciapetti G., Taddei P. (2010). Apatite formation on bioactive calcium-silicate cements for dentistry affects surface topography and human marrow stromal cells proliferation. *Dental Materials*.

[6] Tait C. M. E., Ricketts D. N. J., Higgins A. J. (2005). Restoration of the root-filled tooth: Pre-operative assessment. *British Dental Journal*.

[7] Ioannidis K., Mistakidis I., Beltes P., Karagiannis V. (2013). Spectrophotometric analysis of crown discoloration induced by MTA- and ZnOE-based sealers. *Journal of Applied Oral Science*.

[8] Hakki S. S., Bozkurt S. B., Hakki E. E., Belli S. (2009). Effects of mineral trioxide aggregate on cell survival, gene expression associated with mineralized tissues, and biomineralization of cementoblasts. *Journal of Endodontics*.

[9] Wang X.-H., Li G.-P., Yang W.-S., Jiao Z.-Q., Liu H.-M., Ni Y.-P. (2016). Cardioprotective effects of traditional Chinese medicine Guanmaitong on acute myocardial infarction. *Experimental and Therapeutic Medicine*.

[10] Yu S., Fourman M. S., Mahjoub A. (2017). Lung cells support osteosarcoma cell migration and survival. *BMC Cancer*.

[11] He H., Ren X., Wang X. (2012). Therapeutic effect of Yunnan Baiyao on rheumatoid arthritis was partially due to regulating arachidonic acid metabolism in osteoblasts. *Journal of Pharmaceutical and Biomedical Analysis*.

[12] Dai C., Liang Y., Hao H. (2013). Global detection and identification of components from Yunnan Baiyao based on liquid chromatography hybrid ion trap time-of-flight mass spectrometry. *Journal of Separation Science*.

[13] Liu X. S., Guan X. B., Chen R. Y., Hua H., Liu Y., Yan Z. M. (2012). Repurposing of Yunnan Baiyao as an alternative therapy for minor recurrent aphthous stomatitis. *Evidence-Based Complementary and Alternative Medicine*.

[14] An S., Ling J., Gao Y., Xiao Y. (2012). Effects of varied ionic calcium and phosphate on the proliferation, osteogenic differentiation and mineralization of human periodontal ligament cells in vitro. *Journal of Periodontal Research*.

[15] Annibali S., Cicconetti A., Cristalli M. P. (2013). A comparative morphometric analysis of biodegradable scaffolds as carriers for dental pulp and periosteal stem cells in a model of bone regeneration. *The Journal of Craniofacial Surgery*.

[16] Huang G. T.-J., Sonoyama W., Liu Y., Liu H., Wang S., Shi S. (2008). The hidden treasure in apical papilla: the potential role in pulp/dentin regeneration and bioroot engineering. *Journal of Endodontics*.

[17] He W., Wang Z., Luo Z. (2015). LPS Promote the odontoblastic differentiation of human dental pulp stem cells via MAPK signaling pathway. *Journal of Cellular Physiology*.

[18] Ma S., Liu G., Jin L. (2016). IGF-1/IGF-1R/hsa-let-7c axis regulates the committed differentiation of stem cells from apical papilla. *Scientific Reports*.

[19] Lei G., Yan M., Wang Z. (2011). Dentinogenic capacity: immature root papilla stem cells versus mature root pulp stem cells. *Biology of the Cell*.

[20] Chrepa V., Henry M. A., Daniel B. J., Diogenes A. (2015). Delivery of apical mesenchymal stem cells into root canals of mature teeth. *Journal of Dental Research*.

[21] Wang J., Liu B., Gu S., Liang J. (2012). Effects of Wnt/*β*-catenin signalling on proliferation and differentiation of apical papilla stem cells. *Cell Proliferation*.

[22] Liu H., Wang L., Xiao X. (2008). Anomalous high-pressure behavior of amorphous selenium from synchrotron x-ray diffraction and microtomography. *Proceedings of the National Acadamy of Sciences of the United States of America*.

[23] Ślósarczyk A., Paszkiewicz Z., Paluszkiewicz C. (2005). FTIR and XRD evaluation of carbonated hydroxyapatite powders synthesized by wet methods. *Journal of Molecular Structure*.

[24] Li J., Yan M., Wang Z. (2014). Effects of canonical NF-*κ*b signaling pathway on the proliferation and odonto/osteogenic differentiation of human stem cells from apical papilla. *BioMed Research International*.

[25] Yu J., He H., Tang C. (2010). Differentiation potential of STRO-1+ dental pulp stem cells changes during cell passaging. *BMC Cell Biology*.

[26] Zhao X., He W., Song Z., Tong Z., Li S., Ni L. (2012). Mineral trioxide aggregate promotes odontoblastic differentiation via mitogen-activated protein kinase pathway in human dental pulp stem cells. *Molecular Biology Reports*.

[27] Minamikawa H., Deyama Y., Nakamura K. (2009). Effect of mineral trioxide aggregate on rat clonal dental pulp cells: expression of cyclooxygenase-2 mRNA and inflammation-related protein via nuclear factor kappa B signaling system. *Journal of Endodontics*.

[28] Wan F., Gao L., Lu Y. (2016). Proliferation and osteo/odontogenic differentiation of stem cells from apical papilla regulated by Zinc fingers and homeoboxes 2: an in vitro study. *Biochemical and Biophysical Research Communications*.

[29] Yan M., Wang Z., Zheng Y. (2014). 17 beta-estradiol promotes the odonto/osteogenic differentiation of stem cells from apical papilla via mitogen-actiated protein kinase pathway. *Stem Cell Research & Therapy*.

[30] Olsen J. J., Skov J., Ingerslev J., Thorn J. J., Pinholt E. M. (2016). Prevention of bleeding in orthognathic surgery - A systematic review and meta-analysis of randomized controlled trials. *Journal of Oral and Maxillofacial Surgery*.

[31] Li R., Alex P., Ye M. (2011). An old herbal medicine with a potentially new therapeutic application in inflammatory bowel disease. *International Journal of Clinical and Experimental Medicine*.

[32] Jani P. H., Gibson M. P., Liu C. (2016). Transgenic expression of Dspp partially rescued the long bone defects of Dmp1-null mice. *Matrix Biology*.

[33] Frasheri I., Ern C., Diegritz C., Hickel R., Hristov M., Folwaczny M. (2016). Full-length amelogenin influences the differentiation of human dental pulp stem cells. *Stem Cell Research & Therapy*.

[34] Park S.-J., Lee H.-K., Seo Y.-M., Son C., Bae H. S., Park J.-C. (2018). Dentin sialophosphoprotein expression in enamel is regulated by Copine-7, a preameloblast-derived factor. *Archives of Oral Biolog*.

[35] Huang J., Zhao L., Xing L., Chen D. (2010). MicroRNA-204 regulates Runx2 protein expression and mesenchymal progenitor cell differentiation. *Stem Cells*.

[36] Xu J., Wang B., Sun Y. (2016). Human fetal mesenchymal stem cell secretome enhances bone consolidation in distraction osteogenesis. *Stem Cell Research & Therapy*.

[37] Lin X., Dong R., Diao S. (2017). SFRP2 enhanced the adipogenic and neuronal differentiation potentials of stem cells from apical papilla. *Cell Biology International*.

[38] Bouleftour W., Bouet G., Granito R. N. (2015). Blocking the expression of both bone sialoprotein (BSP) and osteopontin (OPN) impairs the anabolic action of PTH in mouse calvaria bone. *Journal of Cellular Physiology*.

[39] Tang J., Saito T. (2015). Effect of type I collagen derived from tilapia scale on odontoblast-like cells. *Tissue Engineering and Regenerative Medicine*.

[40] He X., Jiang W., Luo Z. (2017). IFN-*γ* regulates human dental pulp stem cells behavior via NF-*κ*B and MAPK signaling. *Scientific Reports*.

[41] Anderson J. D., Johansson H. J., Graham C. S. (2016). Comprehensive proteomic analysis of mesenchymal stem cell exosomes reveals modulation of angiogenesis via nuclear factor-*κ*B signaling. *Stem Cells*.

[42] Wang Y., Yan M., Fan Z., Ma L., Yu Y., Yu J. (2014). Mineral trioxide aggregate enhances the odonto/osteogenic capacity of stem cells from inflammatory dental pulps via NF-*κ*B pathway. *Oral Diseases*.

[43] Coen K., Flannagan R. S., Baron S. (2012). Lysosomal calcium homeostasis defects, not proton pump defects, cause endo-lysosomal dysfunction in PSEN-deficient cells. *The Journal of Cell Biology*.

[44] Tai V., Leung W., Grey A., Reid I. R., Bolland M. J. (2015). Calcium intake and bone mineral density: Systematic review and meta-analysis. *BMJ*.

[45] Ge C., Yu M., Petitte J. N., Zhang C. (2009). Epidermal growth factor-induced proliferation of chicken primordial germ cells: involvement of calcium/protein kinase C and NFKB1. *Biology of Reproduction*.

[46] González Pardo V., D'Elia N., Boland R., Russo de Boland A. (2011). Involvement of the NFKB pathway in 1Α,25(OH)2-vitamin D3-induced growth inhibition of Kaposi sarcoma. *Bone*.

[47] González-Murillo Á., Fernández L., Baena S. (2015). The NFKB inducing kinase modulates hematopoiesis during stress. *Stem Cells*.

